# Faking it - I *The Menace of Counterfeit Drugs*

**DOI:** 10.4103/0019-5545.44743

**Published:** 2008

**Authors:** G. Swaminath

**Affiliations:** Department of Psychiatry, Dr. B. R. Ambedkar Medical College, Kadugondanahalli, Bangalore- 560 045, Karnataka, India

## TRUE LIES

In a blow to the revenues of India's largest pharmaceutical manufacturer and one of the world's leading generic companies, the United States Food and Drug Administration (FDA) has blacklisted about 30 generic drugs being manufactured by the company at two of their manufacturing sites, citing failure to adhere to current Good Manufacturing Practices requirements. The US has not sought a ban nor sought withdrawal of the products manufactured from the blacklisted sites, as the FDA's repeated testing and review led the agency to conclude that there is no reason to question the safety or effectiveness of blacklisted drugs.[1]

There are many who feel that this action by the FDA is unduly harsh and probably protectionist. However, the FDA wants to send a clear signal that drug products intended for use by American consumers must meet their standards of safety and quality.[1] To those around the world who were cursing India for its poor drug regulatory record, the present FDA sanction seems like divine retribution. It is nevertheless unfortunate that the axe has fallen on a company with a reputation for the affordability and safety of its generic drugs.

India has the notoriety of being a major producer of the world's counterfeit drugs. Counterfeit drugs form 10% of the world's drug trade according to the World Health Organisation (WHO). Globally, this trade in fake medicines is one of the fastest growing grey economies – after prostitution, narcotics, terrorism, and arms trade.[2] The Mashelkar Committee,[3] which studied the various aspects of the growing threat from spurious drugs, quoted figures which varied from 0.5% (from state authority figures) to a widely quoted 35%, taken from a report in the *Lancet* in 2001[4] ostensibly based on WHO figures. The average figure over studies is 15–20%, with the annual rate of growth at 25%, and manufacturing concentrated in northern states of India. The WHO, however, clarified it had not conducted any study that had said that 35 per cent of world's spurious drugs are produced in India. It added, “The majority of the Indian pharmaceuticals are produced by large manufacturers according to WHO Good Manufacturing Practices (GMP)!”[5] This kudos from the WHO is gratifying as India also is a leading source of high quality drugs sold by legitimate drug manufacturers, at affordable prices, sometimes at as low as a tenth of the cost in other countries, as is seen with psychiatric medication.

According to the WHO,[2] counterfeit in relation to medicinal products means the deliberate and fraudulent mislabelling with respect to the identity, authenticity, effectiveness, composition and/or source of a finished medicinal product, or ingredient for the preparation of a medicinal product. Counterfeiting can be applicable to both generic and branded products as well as traditional remedies.

## IMITATION: THE BEST FORM OF CHICANERY?

Counterfeit products may include i) products with correct ingredients, but containing insufficient or erroneous quantities of active ingredients, or expired active ingredients either to save cost or owing to poor quality control factors; ii) wrong ingredients with possibly toxic elements and impurities and therefore directly harmful to patients; iii) without active ingredients or using similar class of cheaper ingredients to escape detection; iv) produced by unhygienic manufacture, or lack of rigorous cleaning between production batches; or v) products with false or misleading packaging.[2,6]

The situation is complicated by the fact that counterfeit drugs often contain active pharmaceutical ingredients, if only because the producers are keen to both avoid detection and generate repeat business. The drugs may be ineffective and dangerous as well as difficult to spot because they use active ingredients which are from similar class, for e.g., swapping a patented statin for a cheaper generic form[6] or Chinese manufactured heparin which was contaminated with oversulfated chondroitin sulfate, an inexpensive substance that mimics heparin in basic chemical tests[7] resulting in the death of 95 Americans.

Despite the best efforts of all those crusading against spurious drugs, unfortunately, malevolent dealings in counterfeit drugs are very much a contemporary reality. The modus operandi includes recycling using used vials with intact labels, refilling and re-labelling with packaging similar to branded drugs, imitation, manufacturing without knowledge, reuse beyond expiry date, and large scale counterfeiting.[2] Notorious real examples include neomycin eye drops and meningococcal vaccine made with tap water; paracetamol syrup made with industrial solvent; ampicillin replaced by turmeric; contraceptive pills substituted with wheat flour; and antimalarials, antibiotics, and snake anti-venom containing no active ingredients.[8]

In wealthier countries, the most frequently counterfeited medicines are new, expensive lifestyle medicines, such as hormones, steroids, antihistamines and of course sex performance enhancers like sildenafil. In developing countries, however, the most counterfeited medicines are unfortunately those used to treat life-threatening conditions such as malaria, tuberculosis, and HIV/AIDS.[7]

There is a view that counterfeiting is a crime with no real victims. People desire well-known brands, but at a lower cost, and unscrupulous mercenaries are ready with carefully crafted replicas. With shoes, garments, chocolates, and electronic goods the cheating is relatively innocuous giving the customer the thrill of a ‘great’ product. However, the dangers inherent in fake medication make nonsense of any cozy collusion between a knowing consumer and helpful supplier. Counterfeiting is attractive because relatively small quantities of counterfeit medicines can provide huge profits to the counterfeiter, and is seen to carry less risk than trafficking addictive drugs.[2]

Counterfeiting certainly damages legitimate commerce. It undermines margins of legitimate drug companies, reducing profit for reinvestment in new drugs and for low cost, high quality generics. It reduces the tax revenues of the country, which could be ploughed back in health infrastructure. It also damages reputation of drug companies, demolishes public confidence in medicines, and hence companies are reluctant to publicize incidents of counterfeiting of their products.[7]

Spurious drugs are a cause for concern for all those who are working in health sector such as doctors, pharmacists, nurses and health regulators.[9] The worst affected is of course the consumer who gets entrapped in the web of fake drugs with no respite. India is probably the biggest of all markets for spurious drugs accounting for huge economic losses and untold fatalities. The reason why it is easy to produce and sell fake drugs in India is that laws are lax, judicial system ineffective, the infrastructure for checks woefully inadequate, and this is compounded by lack of personnel involved in supervision and widespread corruption. This attracts the drug producers who sell the medicines cheaply to chemists (or even to practitioners who dispense drugs themselves), as they are only pleased to sell these drugs for big profits involved.

There are 20,000 registered brands as well as an approximate of around 1300 molecules in India as per the Indian Drug Review (IDR) Drug compendium, 2008,[9] but the number of unregistered brands could be double this figure, There are a around 20,000 manufacturers[4] many of them small manufacturers licensed to manufacture generic drugs. There could well be an equal number of unlicensed manufacturers producing generics. The total number of retailers in India at present is around 800,000 distributed among major organized players who form a very small group (1.5%) and the small retailer who forms the majority.[10] Contrast this with developing countries where there is but one brand and a couple of generics after the patent period lapses, and most of the players are organized. The vast number of manufacturers, distributors and retailers makes regulation even more difficult.[10]

## DECEIT MOST FOUL

Most of the spurious drugs find their way into the government institutions like defense and civil hospitals and dispensaries, which bulk purchase drugs through the tender system and settle for the lower bids. The presence of corrupt purchase officials in these deals, the absence of any appropriate system of monitoring the quality of drug, aids spurious and sub-standard drugs finding their way into these places.[2] These spurious drugs unfortunately find their way into the health care system patronized by the poor and illiterate who unfortunately do not have the wherewithal to probe and identify spurious drugs and meekly accept whatever is prescribed or dispensed. The ultimate loser is the patient who fails to improve; a notch below is the doctor, whose reputation would be compromised.[11]

Internet-based sales of pharmaceuticals, which should have been a boon to those seeking cheaper, stigmatized or unauthorized treatments, has unfortunately now become a major source of counterfeit medicines.[7] Some internet pharmacies are completely legal operations, set up to offer clients convenience and savings. They require patient prescriptions and deliver medications from government licensed facilities.[7] However, illegal internet pharmacies sell medications without prescriptions and use unapproved or counterfeit products. In some cases, internet pharmacies are operated internationally and sell products that have an unknown or vague origin.[7]

Non-response of illness to treatment prompts physicians to test out an algorithm of reasons. This algorithm in its list has inadequate dosage as one of the causes. It is prudent to consider spurious drugs in this algorithm, given the enormity of the problem. Failure to consider this may cause change of diagnosis with an unnecessary battery of tests to clarify it.[2] Further, in case of toxic reactions, which could unfortunately sometimes be fatal, the generic drug could be labeled dangerous, and consequently avoided.[2]

The Indian regulatory system has dual responsibility a) protecting Indians from the menace of spurious and substandard drugs and b) preparing Indian companies to meet the highest standards of the global market. It is high time the regulatory system works. It is when the existing laws are reinforced uniformly and stringently, and if the guilty manufacturers, up to those involved in dispensing are caught and punished accordingly, this menace can be curbed to a large extent. The end users of the drugs, namely, the doctors and the patients should be educated and made aware of the problem, and they should also take responsibility in curbing the menace. This will be discussed in the next issue.

(Note: Spurious, substandard, fake and counterfeit drugs have been used interchangeably despite having differences in definition, as the focus is on the hazards to the consumer).

## References

[1] http://www.thehindubusinessline.com/2008/09/18/stories/2008091852700100.htm.

[2] Jain SK (2006). The spurious drug menace and remedy. Health Administrator.

[3] Mashelkar Committee Report (2003). Ministry of Health and Family Welfare, Government of India.

[4] Chatterjee P (2001). India's trade in fake drugs-bringing the counterfeiters to book. Lancet.

[5] Ramachandran R Dealing with fake drugs.

[6] Jack A (2007). Bitter pills: Counterfeit Medicines. BMJ.

[7] WHO Substandard and counterfeit medicines http://www.who.int/mediacentre/factsheets/fs275/en/.

[8] Newton P (2002). Murder by fake drugs, Time for international action. BMJ.

[9] (2008). IDR Drug triple I compendium.

[10] (2008). Booming Pharma Sector in India, Market Research Reports, Marketing and Business Research, Industry Analysis.

[11] Dhikav V Re: Spurious drugs for real money.

